# Faecal immunochemical test accuracy in patients referred for surveillance colonoscopy: a multi-centre cohort study

**DOI:** 10.1186/1471-230X-12-94

**Published:** 2012-07-24

**Authors:** Jochim S Terhaar sive Droste, Sietze T van Turenhout, Frank A Oort, René WM van der Hulst, Vincent A Steeman, Usha Coblijn, Lisette van der Eem, Ruud Duijkers, Anneke A Bouman, Gerrit A Meijer, Annekatrien CTM Depla, Pieter Scholten, Ruud JLF Loffeld, Veerle MH Coupé, Chris JJ Mulder

**Affiliations:** 1 Gastroenterology and Hepatology, VU University Medical Centre, Amsterdam, The Netherlands; 2 Gastroenterology and Hepatology, Kennemer Gasthuis, Haarlem, The Netherlands; 3 Clinical Chemistry, VU University Medical Centre, Amsterdam, The Netherlands; 4 Pathology, VU University Medical Centre, Amsterdam, The Netherlands; 5 Gastroenterology and Hepatology, Slotervaart Hospital, Amsterdam, The Netherlands; 6 Gastroenterology and Hepatology, Sint Lucas Andreas Hospital, Amsterdam, The Netherlands; 7 Internal Medicine, Zaans Medical Centre, Zaandam, The Netherlands; 8 Epidemiology and Biostatistics, VU University Medical Centre, Amsterdam, The Netherlands; 9 Department of Gastroenterology and Hepatology, VU University Medical Centre, P.O. Box 7057, 1007 MB Amsterdam, The Netherlands

**Keywords:** Colorectal cancer, Faecal immunochemical test (FIT), Surveillance, Advanced adenoma, Sensitivity

## Abstract

**Background:**

Given the increasing burden on colonoscopy capacity, it has been suggested that faecal immunochemical test (FIT) results could guide surveillance colonoscopy intervals. Against this background, we have evaluated the test accuracy of single and double FIT sampling to detect colorectal cancer (CRC) and/or advanced adenomas in an asymptomatic colonoscopy-controlled high-risk population.

**Methods:**

Cohort study of asymptomatic high-risk patients (personal history of adenomas/CRC or family history of CRC), who provided one or two FITs before elective colonoscopy. Test accuracy of FIT for detection of CRC and advanced adenomas was determined (cut-off level 50 ng/ml).

**Results:**

1,041 patients provided a FIT (516 personal history of adenomas, 172 personal history of CRC and 353 family history of CRC). Five CRCs (0.5%) and 101 advanced adenomas (9.7%) were detected by colonoscopy. Single FIT sampling resulted in a sensitivity, specificity, PPV and NPV for CRC of 80%, 89%, 3% and 99.9%, respectively, and for advanced adenoma of 28%, 91%, 24% and 92%, respectively. Double FIT sampling did not result in a significantly higher sensitivity for advanced neoplasia. Simulation of multiple screening rounds indicated that sensitivity of FIT for advanced adenoma could reach 81% after 5 screening rounds.

**Conclusions:**

In once-only FIT sampling before surveillance colonoscopy, 70% of advanced neoplasia were missed. A simulation approach indicates that multiple screening rounds may be more promising in detecting advanced neoplasia and could potentially alleviate endoscopic burden.

## Background

The use of colonoscopy to evaluate patients for the presence of colorectal cancer (CRC) has increased substantially in the past decade [1-3]. This concerns both high-risk asymptomatic individuals and patients with signs or symptoms suggestive of CRC. At present, 22% of colonoscopies in the United States are performed for a family history of CRC and another 22% for surveillance after polypectomy or resection of CRC [4]. In The Netherlands, 33% of colonoscopies are performed for a personal history of adenomas or CRC or a family history of CRC [5]. With the planned introduction of a CRC screening program in 2013 in The Netherlands, not only the number of screening colonoscopies, but also the number of surveillance colonoscopies because of a personal history of colorectal neoplasia will increase [6]. Since the burden on endoscopic capacity is already high in some countries, it has been proposed to use faecal immunochemical tests (FITs) to postpone elective colonoscopy in asymptomatic high-risk subjects [7,8]. Previous studies on guaiac-based faecal occult blood tests (g-FOBTs) have shown that the clinical use of this strategy was hampered by its relatively low sensitivity for advanced adenomas [9]. For that reason, high-risk subjects are recommended not to rely on g-FOBT screening, but to undergo colonoscopic evaluation at predetermined intervals [10-12]. FIT, however, has been proven to be superior to g-FOBTs in detecting advanced adenomas and CRC [13-20]. The higher sensitivity of FIT for advanced adenomas is particularly relevant since the goal of a surveillance program is to detect advanced adenomas before malignant transformation and in this way prevent CRC related death. A recent study in asymptomatic high-risk patients, showed sensitivities of 100% and 65% for the detection of CRC and all advanced neoplasia, respectively [7]. These results need confirmation in a different set of patients, preferably with a larger sample size and higher yield of advanced neoplasia, before contemplating on its use in surveillance programs. The present study evaluates the test accuracy of single and double FIT sampling to detect CRC and/or advanced adenomas in a colonoscopy-controlled high-risk population.

## Methods

### Study population and study design

All consecutive patients scheduled for elective colonoscopy from June 2006 to October 2009 at one of the five participating hospitals, were invited to participate in the original study [16]. Details of study design and of most materials and methods relevant for this study have been published previously [16]. In addition to the original study protocol in which subjects were asked to provide one FIT, we extended the protocol by asking for a second FIT sample before colonoscopy in the final 15 months of inclusion (June 2008 to October 2009) [21]. For the present study, all asymptomatic patients with a personal history of adenomas/CRC or asymptomatic individuals with a family history of CRC who underwent complete colonoscopy and who performed at least one FIT were analyzed retrospectively. In all centres, local Medical Ethics Review Board approval was obtained prior to the start of the study.

**Figure 1:**
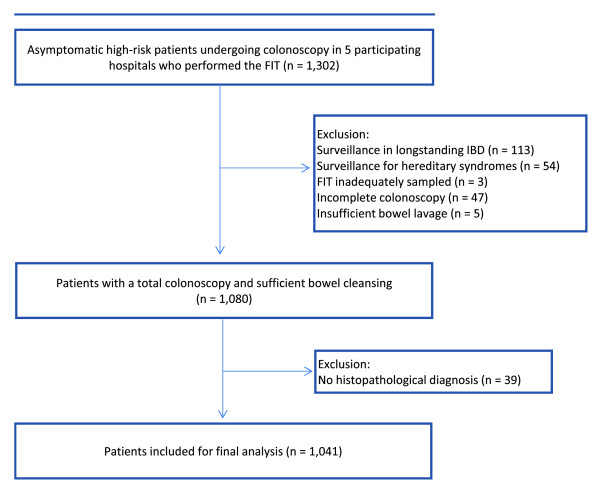
Study flow diagram.

All eligible individuals were asked to provide one or two FITs on stool from one or two bowel movements prior to colonoscopy. Exclusion criteria are shown in Figure 1.

All medical files were assessed to evaluate the time interval since prior colonoscopy, previous presence of advanced neoplasia, cumulative number, size and histology of polyps detected at previous colonoscopies, and a self-reported two-generation family history of CRC. A significant family history of CRC was defined as one first-degree relative with CRC diagnosis under the age of 60 years or with more than one first-degree relative with CRC of any age [22]. A non-significant family history was defined as one first-degree relative older than 60 years with a CRC diagnosis or second-degree relatives with a CRC diagnosis.

### Faecal immunochemical test

**Figure 2:**
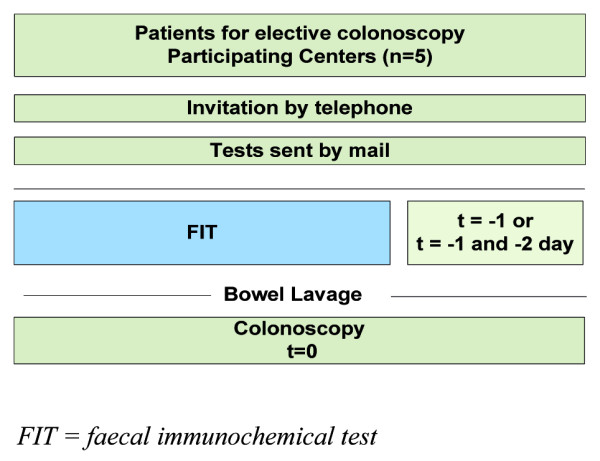
Flowchart of invitation and FIT sampling.

The FIT used in the present study is the automated OC-sensor test (Eiken Chemical Co., Tokyo, Japan), which has a quantitative outcome. The baseline FIT was performed on a bowel movement one day before colonoscopy (t = –1), whereas the additional FIT for double sampling was performed on stool obtained two days prior to colonoscopy (t = –2; see Figure 2) (at maximum 72 hours prior to colonoscopy). Both FITs were performed before bowel preparation had started. Patients who performed the FIT after starting bowel preparation were excluded. Illustrated instructions guided the participants to sample their stool ensuring that contact with water and urine was prevented. No restrictions were made with regard to diet during the week in which the stool sample was taken [23]. On the day of colonoscopy, the completed test and the signed informed consent form were handed over to the nursing staff at the endoscopy department. All FITs were stored at minus 5 degrees Celsius on arrival. Individuals were requested to keep the FIT refrigerated before handing in the test at the endoscopy department. Tests were analyzed using the OC sensor MICRO desktop analyzer (Eiken Chemical Co., Tokyo, Japan) according to the manufacturers instructions [24]. For single FIT sampling a cut-off level of 50 ng/ml was used. For double FIT sampling, we considered the FIT positive when the cut-off level exceeded 50 ng/ml in at least one out of two samples. In general, tests were analyzed within one week by one of two experienced technicians who were unaware of the clinical data. In case tests could not be analyzed within one week, the tests were deep frozen at minus 20 degrees Celsius. Both technicians received special training for analyzing the tests.

### Standards of reference

Colonoscopy was the standard of reference for the presence, size and location of colorectal neoplasia. Colonoscopies were performed or supervised by experienced gastroenterologists. Endoscopists were blinded to the FIT result. Conscious sedation using Midazolam was offered to all patients. A complete colonoscopy was defined as intubation of the cecum with identification of the ileocecal valve or appendiceal orifice, or intubation up to an obstructing CRC. The results of histopathological analysis of tissue samples obtained during colonoscopy were the standard of reference for the diagnosis of adenoma or cancer. Adenomas ≥1.0 cm, with any villous features (i.e. tubulovillous or villous adenoma) or high-grade dysplasia, were considered advanced adenomas [25,26]. Advanced neoplasia included all cases of CRC and all advanced adenomas. If multiple lesions were present, classification was based on the most advanced lesion found.

Adequacy of adherence to surveillance guidelines was evaluated by comparing the actual surveillance intervals in this population to the national and/or international guidelines [27,28].

### Statistical analysis

Taking colonoscopy as the reference test, sensitivity, specificity, positive and negative predictive value (PPV and NPV) of FIT were calculated for the following colonoscopy outcomes: 1) the presence of CRC; 2) the presence of advanced adenoma; and 3) the presence of advanced neoplasia. Sensitivity was calculated as the proportion of positive test results in patients with the colonoscopy outcome under consideration. Specificity was calculated as the proportion of negative test results in patients with an outcome less severe than the colonoscopy outcome under consideration. Note that, therefore, the same specificity results from choosing either outcome 2 or 3. For dichotomizing the FIT results, a cut-off level of 50 ng/ml was used, which is considered the most sensitive cut-off level for the OC-sensor. The 95% confidence intervals (CI) were calculated using the exact method (http://www.measuringusability.com/wald.htm).

The Chi-square test was used for the comparison of proportions. A two sided P*-*value of < 0.05 was considered statistically significant. All analyses were performed with SPSS for Windows Version 15 (SPSS Inc., Chicago, Illinois).

### Modelling cumulative sensitivity after multiple rounds of FIT screening

This study was not designed to provide information on interval FIT testing in a surveillance program. Given the fact that sensitivity of FIT for advanced adenomas is moderate, we explored the potential of repeated testing rounds by modelling cumulative sensitivity as has been described previously [29]. In brief, this model assumes unbiased detection each time testing is done and cumulative sensitivity can be calculated with the following formula: (1-(1-sensitivity)^x^). The x variable represents the number of FIT testing rounds.

## Results

### Characteristics of the study population

Overall 1,041 asymptomatic high-risk subjects who underwent surveillance colonoscopy provided one FIT. A subgroup of 43% of these patients provided two FITs (n = 451). The mean age of all patients was 60.7 years (range 27–87 years) and 50.1% of these were male. Colonoscopy was performed because of a personal history of adenomas in 516 patients (49.6%). In 172 patients (16.5%) and 353 patients (33.9%), colonoscopy was performed because of a personal history of CRC and a family history of CRC, respectively. In 249/1041 patients (23.9%) a significant family history of CRC was observed. The date and findings of the last colonoscopy prior to FIT sampling were known for 872/1041 patients (84%). In 305 patients the previous colonoscopy was within two years of the current colonoscopy and FIT. A total of 386 patients had a colonoscopy more than two years prior to their current colonoscopy and FIT. In 181 patients with a family history of CRC, the current colonoscopy was their first colonoscopy. In 21% (147/691) of patients for whom the findings of the last colonoscopy were known, the recommended guidelines for surveillance were followed correctly. In 63% (434/691) of patients, surveillance colonoscopy was performed earlier than the recommended interval.

### Colonoscopy results

CRC was found in five patients (0.5%). Of these, three were found in patients with a personal history of CRC, one in a patient with a family history of CRC and one in a patient with a personal history of adenomas. Early stage CRC (AJCC stage I/II) was found in 3/5 patients (60%) and in 2/5 patients (40%) the tumour was located in the proximal colon. Two patients with recurrent CRC had a previous colonoscopy two years before. In the third case with recurrent CRC, no data on the time interval could be retrieved. In one patient with a family history of CRC, the CRC diagnosis was obtained during his first colonoscopy. In the CRC patient with a personal history of adenomas, only two small adenomas were found during previous colonoscopy. In 101 patients (9.7%) at least one advanced adenoma was found. Of these, 19 (19%) were found in patients with a personal history of CRC, 23 (23%) in patients with a family history of CRC and 59 (58%) in patients with a personal history of adenomas. In 47/101 patients (47%), the advanced adenoma was found in the proximal colon. Of all advanced adenomas, 42% had >3 adenomas diagnosed on previous colonoscopies, 67% (n = 68) were sized >10 mm, 28% (n = 28) had villous histology and 5% (n = 5) had high grade dysplasia. Advanced neoplasia (CRC and advanced adenoma together) were found in 106 patients (10.2%). The yield of advanced neoplasia per indication group is shown in Table 1. The quality of bowel preparation was rated “good” in 77% (n = 805) of colonoscopies and was rated “fair” in 23% (n = 236) of colonoscopies. The cecal intubation rate was 96.4% (see Figure 1). No serious procedure related complications were observed.

**Table 1:** Demographics and yield of advanced neoplasia‡ in three different indication groups in 1041 asymptomatic, high-risk subjects referred for surveillance colonoscopy

		**N (%)**	**Demographics**		**Yield**		
			**Mean age (range)**	**Males (%)**	**CRC (%)**	Adv Adenomas*(%)	**Advanced neoplasia (%)**
Indication for colonoscopy	Personal history of adenomas	516 (49.6)	63.2 (29–87)	279 (54.1)	1 (0.2)	59 (11.4)	60 (11.6)
	Personal history of CRC^*§*^	172 (16.5)	66.2 (44–86)	82 (47.7)	3 (1.7)	19 (11.0)	22 (12.8)
	Family history of CRC^*§*^	353 (33.9)	54.3 (27–82)	161 (45.6)	1 (0.3)	23 (6.5)	24 (6.8)
	Total population	1041 (100)	60.7 (27–87)	522 (50.1)	5 (0.5)	101 (9.7)	106 (10.2)

‡ *Advanced neoplasia is defined as either one or more advanced adenoma(s) or a CRC.*

§ CRC: colorectal cancer.

* Advanced adenoma: Adenomas ≥1.0 cm, with any villous features (i.e. tubulovillous or villous adenoma) or high-grade dysplasia.

### FIT results

The overall FIT positivity rate in single FIT sampling was 11% (115/1041 patients). The positivity rate for the subgroup of patients that provided two FITs was 19% (87/451 patients). Table 2 summarizes the test accuracy of single and double FIT sampling for the presence of CRC and advanced adenomas. The overall sensitivity for CRC and advanced neoplasia in the total population was 80% and 30% respectively, missing 1 out of 5 cancers and 74 out of 106 advanced neoplasia. Double FIT sampling resulted in a sensitivity of 33% for advanced neoplasia, missing 28 out of 42 advanced neoplasia. Sensitivity for patients with left- versus right-sided advanced neoplasia was 38% (20/53) and 22% (11/47), respectively. The positivity rates and test accuracy of single FIT sampling for the presence of all advanced neoplasia in the three indication groups separately are shown in Table 3. The positivity rate was significantly higher both in patients with a personal history of CRC and patients with a personal history of adenomas compared to patients with a family history of CRC (12%; n = 21/172 and 14%; n = 70/516 versus 6.8%; n = 24/353; p = 0.04 and p = 0.002).

**Table 2:** Test characteristics of single and double FIT sampling (OC-sensor) for colorectal cancer and advanced adenoma at a cut-off level of 50 ng/ml in asymptomatic, high-risk subjects referred for surveillance colonoscopy

	**Single FIT*(n*** **= 1041)**		**Double FIT (one FIT+)*(n*** **= 451)**	
	**CRC***§ ****(n*** **= 5)**	**Advanced adenoma**‡ **(*n*** **= 101)**	**CRC***§* (***n*** **= 2)**	**Advanced adenoma**‡ ***(n*** **= 40)**
**Sensitivity**	**80%**	**28%**	**50%**	**33%**
N	4/5	28/101	1/2	13/40
(CI in %)	(28–99)	(19–38)	(1–99)	(19–49)
**Specificity**	**89%**	**91%**	**81%**	**82%**
N	925/1036	852/935	363/449	336/409
(CI in %)	(87–91)	(89–93)	(77–84)	(78–86)
PPV*	**3%**	**24%**	**1%**	**15%**
N	4/115	28/115	1/87	13/87
(CI in %)	(1–9)	(17–33)	(0,03–6)	(8–24)
**NPV**†	**99,9%**	**92%**	**99,7%**	**93%**
N	925/926	853/926	363/364	337/364
(CI in %)	(99–100)	(90–94)	(98–99,9)	(89–95)

* PPV: positive predictive value.

*† NPV: negative predictive value*.

*§ CRC: colorectal cancer*.

*‡ Advanced adenoma: Adenomas ≥1.0 cm, with any villous features (*i.e. *tubulovillous or villous adenoma) or high-grade dysplasia*.

**Table 3:** Positivity rates and test characteristics of single FIT sampling for advanced neoplasia‡ in three different indication groups in 1041 asymptomatic, high-risk subjects referred for surveillance colonoscopy

		**Indication groups**			
		**Personal history of adenomas (n = 516)**	**Personal history of CRC§ (n = 172)**	**Family history of CRC§ (n = 353)**	**Total (n = 1041)**
**Test characteristics**	**Positivity rate**	**14%**	**12%**	**7%**	**11%**
	N	70/516	21/172	24/353	115/1041
	**Sensitivity**	**32%**	**36%**	**21%**	**30%**
	N	19/60	8/22	5/24	32/106
	(CI in %)	(20–45)	(17–59)	(7–42)	(22–40)
	**Specificity**	**89%**	**91%**	**94%**	**91%**
	N	405/456	137/150	310/329	852/935
	(CI in %)	(86–92)	(86–95)	(91–96)	(89–93)
	PPV*	**27%**	**38%**	**21%**	**28%**
	N	19/70	8/21	5/24	32/115
	(CI in %)	(17–39)	(18–62)	(7–42)	(20–37)
	**NPV**†	**91%**	**91%**	**94%**	**92%**
	N	405/446	137/151	310/329	852/926
	(CI in %)	(88–93)	(85–95)	(91–96)	(90–94)

‡ *Advanced neoplasia is defined as either one or more advanced adenoma(s) or a CRC*.

* PPV: positive predictive value.

*† NPV: negative predictive value*.

*§ CRC: colorectal cancer*.

Regarding all test characteristics, only specificity for CRC and specificity for advanced adenoma differed significantly between the patients with a personal history of colorectal neoplasia and patients with a family history of CRC (specificity for CRC is 87.1%; n = 596/684 versus 93.5%; n = 329/352; p = 0.002; specificity for advanced adenoma is 89.4%; n = 542/606 versus 94.2%; n = 310/329; p = 0.014). No significant differences in specificity for CRC or advanced adenomas were found when separating personal history of neoplasia in personal history of CRC and personal history of adenomas. No significant differences in sensitivity, PPV and NPV for either CRC or advanced adenoma were found between these three groups. Sensitivity of FIT for detecting all advanced neoplasia was not significantly higher in patients with a significant family history for CRC compared to patients with a non-significant family history of CRC (33.3%; n = 3/9 versus 13.3%; n = 2/15; p = 0.33).

### Cumulative sensitivity after multiple rounds of FIT screening

A once-only FIT sensitivity of 80% for CRC compounds to a sensitivity of 99.2% with 3 rounds of testing (1-(1-sensitivity)^3^). Similarly for advanced adenoma detection, modelling a 28% once-only sensitivity compounds over 3 rounds of testing to 63%, and over 5 rounds of testing to a sensitivity of 81%.

## Discussion

In the present study, test accuracy of one of the most commonly used FITs was evaluated in a large cohort of asymptomatic, high-risk individuals undergoing colonoscopy surveillance. It was found that 20% of CRCs and 72% of advanced adenomas were missed when using a single FIT sampling strategy at the lowest cut-off level. Moreover, providing a second FIT preceding colonoscopy did not result in a significant increase in sensitivity since still only 33% of all advanced neoplasia was detected. When the indication groups were separated into patients with a personal history of CRC, a personal history of adenomas and patients with a family history of CRC, a higher FIT positivity rate and a higher yield of advanced neoplasia was observed in the first two groups. Sensitivity, however, did not significantly differ between these three indication groups, showing that a once-only FIT sampling strategy is inadequate in detecting advanced neoplasia in either group of high-risk, asymptomatic individuals.

Limited data are available on the performance of FITs in asymptomatic high-risk patients. Three studies have assessed the efficacy of a first generation, qualitative FIT (OC light and Hemeselect) in volunteer first-degree relatives of patients with CRC, where sensitivities ranged from 50–83% for detection of advanced neoplasia [8,30,31]. However, small sample sizes and small numbers of target lesions hamper the generalization of these results. In patients with a personal history of CRC, one study showed a 100% sensitivity for detecting 9 recurrent CRCs [32]. These results were obtained from stools collected with digital examination and therefore not representative for at home FIT sampling. Moreover, test characteristics for advanced adenomas were not available. Another study, using a qualitative FIT in a larger population, showed sensitivities of 70% and 44% for detecting CRC and advanced adenoma, respectively [33]. Yet, incorrect criteria for defining advanced adenoma were used. In general, test characteristics from qualitative FITs should be interpreted with caution since the test performance can vary greatly between the different qualitative FITs, indicating that quality assurance is an issue [34].

The most promising results were shown in a recent study, providing three FITs preceding colonoscopy in asymptomatic high-risk patients [7]. Sensitivities of 100% and 65% for the detection of CRC and all advanced neoplasia, respectively, were found using the same FIT as was used in the present study at the same cut-off level [7]. Unfortunately, we were not able to confirm these promising findings in our study with a comparable sample size and a larger number of target lesions. Multiplicity of testing (three versus two FIT samples) and different prevalences of advanced adenomas (higher in the present study) may partly explain the differences between the two studies in FIT performance. Other potential explanations might be that the study by *Hazazi* et al. had a better defined high-risk population with more CRCs located in the distal colon or larger pedunculated advanced adenomas with a higher tendency to bleed or a better quality of bowel preparation since 23% of colonoscopies was rated “fair” bowel preparation in the present study. Moreover, in the present study a high percentage of patients have received their colonoscopy at an earlier time interval than recommended by the guidelines which might have resulted in smaller sized advanced adenomas.

Periodically performing non-invasive FIT sampling in the surveillance of high-risk individuals in order to triage individuals for invasive colonoscopy sounds appealing, as this could lead to a better utilization of colonoscopy as both a diagnostic and therapeutic procedure. However, when considering such an alternative surveillance scheme, a high sensitivity of FIT is important. Specificity is less of a concern, because at present colonoscopy is performed in all these patients. Even at the most sensitive cut-off level of 50 ng/ml in single and double FIT sampling, the majority of advanced neoplasia was missed in the present study. On the one hand it is debatable whether missing advanced adenomas is clinically relevant, since they could be detected in subsequent surveillance rounds (either with FIT or colonoscopy) while still being in a curable stage. On the other hand, a delay in detecting CRC can result in progression into advanced stage disease. Yet, the risk of progression from adenoma to carcinoma in the majority of patients with a family history of CRC and in the majority of patients with a personal history of adenomas may be limited and comparable to the risk in the general population [10,35,36]. Particularly in patients with only a few small tubular adenomas in the past or a non-significant family history of CRC, multiple rounds of FIT sampling may be a good alternative to colonoscopic surveillance. The present study was not designed to provide information on interval FIT testing in a surveillance program. Recent data from Australia showed that multiple rounds of FIT sampling within an existing surveillance program aided the detection of advanced neoplasia [29]. In that particular study, it was clearly demonstrated that repeated testing results in a compounding of sensitivity. It was also shown that in those patients who returned a negative FIT in multiple rounds of testing, the chance of finding advanced neoplasia was significantly reduced [29]. When we model cumulative detection in the same manner it can be calculated that a once-only FIT sensitivity of 80% for CRC compounds to 99.2% with 3 rounds of testing. Similarly for advanced adenoma detection, modelling a 28% once-only sensitivity compounds over 5 rounds of testing to 81%, which may be acceptable for the detection of an advanced adenoma. Although these computed sensitivities should be interpreted with caution, it allows us to explore the effect of multiple rounds of testing. Such a strategy of repeated FIT sampling in selected individuals may hold potential to postpone invasive colonoscopy and create more colonoscopic capacity. The effects of earlier diagnosis, particularly of advanced adenomas, on survival remain elusive and might only be clarified in a long-term prospective randomized trial. Since the yield of CRC is generally low in surveillance programs, it is questionable whether such a study is feasible.

There are some concerns that need to be addressed for proper interpretation of our results. First, the number of target lesions is relatively low. Although this study has a large sample size and the percentage of target lesions is higher than in most other studies, the absolute number of cancers is low [7,8,30-33]. Potential explanations for the low number of CRCs may be over-usage of colonoscopy surveillance and inappropriate colonoscopy referral for patients with a non-significant family history of CRC [27,28]. Second, there was substantial heterogeneity in the group of patients with a personal history of adenomas and a family history of CRC, making it difficult to draw firm conclusions for each subgroup.

## Conclusion

The present study of high-risk individuals for colorectal neoplasia, shows that 67–70% of all advanced neoplasia are not detected by single or double FIT sampling before elective colonoscopy. Future studies should focus on multiple rounds of FIT sampling in different, high-risk patient groups in order to create a sensitive strategy to detect advanced neoplasia and potentially alleviate the burden on endoscopic capacity.

## Abbreviations

FIT, Faecal immunochemical test; CRC, Colorectal cancer; PPV, Positive predictive value; NPV, Negative predictive value; g-FOBT, Guaiac-based faecal occult blood test; IBD, Inflammatory bowel disease; NSAID, Non-steroidal anti inflammatory drugs; AJCC, American joint committee on cancer; OR, Odds ratio; CI, Confidence interval; UK, United Kingdom.

## Competing interests

### Declaration of funding interests

This research project was supported by an unrestricted grant of Nycomed BV, Hoofddorp to “The Amsterdam Gutclub”, The Netherlands. This company had no influence on any aspect relevant to this study.

The OC sensor MICRO desktop analyzer was provided by Eiken Chemical co., Tokyo, Japan. This company had no influence on any aspect relevant to this study.

### Declaration of personal interests

None. There were no conflicts of interest for the authors of this study.

## Authors’ contributions

JTSD was responsible for data analysis, interpretation of the data and drafting of the article. SVT was responsible for interpretation of the data and drafting of the article. FO was responsible for interpretation of the data and for critical revision of the article for important intellectual content. RVDH was responsible for conception and design and for critical revision of the article for important intellectual content. VS was responsible for data acquisition and data analysis. UC was responsible for data acquisition and data analysis. LVDE was responsible for data acquisition and data analysis. RD was responsible for data acquisition and data analysis. AB was responsible for analysing the FIT results (data analysis). GM was responsible for conception and design and for critical revision of the article for important intellectual content. AD was responsible for data acquisition and critical revision of the article for important intellectual content. PS was responsible for data acquisition and critical revision of the article for important intellectual content. RL was responsible for data acquisition and critical revision of the article for important intellectual content. VC was responsible for the statistical analysis and for critical revision of the article for important intellectual content. CM was responsible for conception and design and for critical revision of the article for important intellectual content. All authors read and approved the final manuscript.

## Pre-publication history

The pre-publication history for this paper can be accessed here:

http://www.biomedcentral.com/1471-230X/12/94/prepub
